# Content and Dietary Exposure Assessment of Toxic Elements in Infant Formulas from the Chinese Market

**DOI:** 10.3390/foods9121839

**Published:** 2020-12-10

**Authors:** Chuanyou Su, Nan Zheng, Yanan Gao, Shengnan Huang, Xue Yang, Ziwei Wang, Hongjian Yang, Jiaqi Wang

**Affiliations:** 1 Ministry of Agriculture-Laboratory of Quality and Safety Risk Assessment for Dairy Products (Beijing), Institute of Animal Science, Chinese Academy of Agricultural Sciences, Beijing 100193, China; suchuanyou2010@126.com (C.S.); zhengnan@caas.cn (N.Z.); gyn758521@126.com (Y.G.); hsnigot7@163.com (S.H.); yangxue234723@126.com (X.Y.); wswzw12@163.com (Z.W.); 2 College of Animal Science and Technology, China Agriculture University, Beijing 100193, China; yang_hongjian@cau.edu.cn

**Keywords:** infant formula, toxic elements, risk assessment, target hazard quotient

## Abstract

In this study, the content of chromium (Cr), arsenic (As), cadmium (Cd) and lead (Pb) in domestic and imported infant formulas from Beijing, China were analyzed using inductively coupled plasma mass spectrometry. The content of Cr, As, Cd and Pb was 2.51–83.80, 0.89–7.87, 0.13–3.58 and 0.36–5.57 μg/kg, respectively. Even though there were no significant differences in toxic elements content between domestic and imported infant formulas, Cd content was slightly lower in domestic samples. The estimated daily intake (EDI), target hazard quotient (THQ) and hazard index (HI) were calculated for infants between 0.5 and 5 y of age. The EDIs were lower than the oral reference doses. THQ of As, Cr, Cd and Pb was 0.027–0.103, 0.024–0.093, 0.0025–0.0090 and 0.0015–0.0046, respectively. HI values were 0.055–0.192 for boys and 0.056–0.209 for girls and were inversely associated with age with a threshold < 1. The non-carcinogenic risk value were in the safe range, indicating that exposure of As, Pb, Cr and Cd from infant formulas do not represent a health risk in China.

## 1. Introduction

The optimum nutrition for newborns is breastmilk. However, for the past two decades, approximately 67.0% of infants are not completely breastfed for the recommended six months [1]. In China, only 20.8% are completely breastfed for six months, 11.5% are breastfed for one year and 6.9% are breastfed for two years [2]. Infant formulas play a special role in infant nutrition because they can be used as a breastmilk substitute [3]. Suitable infant formulas are preferred sources of nutrition for most infant when breastmilk is absent or insufficient [4]. There are a variety of infant formulas in the Chinese market. In 2017, the import of infant formulas was substantial (296,014 t, $3.98 billion) [5].

According to the International Agency for Research on Cancer, arsenic (As), cadmium (Cd) and hexavalent chromium (Cr VI) are group 1 carcinogens (2012), inorganic lead (Pb) is a group 2A carcinogen (2006) [6]. Epidemiological and experimental evidence indicates that combination effects generated by multi-heavy metals might be quite different from that induced by the same individual metal, because heavy metals at individual low acting concentrations can elicit higher toxicity on interactions [7,8,9]. Pandya et al. [7] showed that under similar dosages, when Pb and Cd are present together, the toxic effect is antagonized by co-exposure due to possible competition among Pb and Cd for hepatic accumulation. Su et al. [10] found that combination of different heavy metals showed certain adverse effects on the hematologic, hepatic, renal and neurobehavioral function and could also disturb electrolyte and lipid balance in rats. This suggests that joint toxicity or interaction patterns among different heavy metals should be taken into consideration during the risk assessment for the exposure to multi-heavy metal simultaneously. The gut immune barrier is the first line of defense against any potentially harmful agents that have been ingested in food [11]. Contrary to the restricted macromolecular passage in adulthood, enhanced transfer takes place across the intestines during early life, due to the high endocytic capacity of the immature intestinal epithelial cells during the neonatal periods [12]. Young individuals are particularly sensitive to chemical contaminants due to their high exposure-body weight ratio [13], immunodeficiency and intestinal hypoplasia. Compared to adults, children have a greater risk to heavy metal exposure through milk consumption [14,15]. Long-term exposure to these toxic elements may cause significant health problems.

To protect public health, maximum levels (MLs) for toxic elements have been set by international organizations and China. The ML of Pb in foods for infant and young children has been set at 150 μg/kg by China [16], 10 μg/kg by Codex Alimentarius Commission (CAC) [17] and 50 μg/kg by the European Union, which set the ML of Cd at 10 μg/kg [18].

Based on the toxicity, frequency of occurrence and potential for human exposure, the Agency for Toxic Substances and Disease Registry developed a substance priority list. According to this list, As, Pb, Cd and Cr are the 1st, 2nd, 7th and 17th priority food contaminants, respectively [19]. In China, toxic elements in food have to be measured to ensure food safety [20]. Therefore, it is of utmost importance to measure the content of toxic elements in infant formulas [21,22]. A previous study reported that certain milk powders and infant formulas are contaminated with toxic elements [21,23]. Recently, toxic elements in infant formulas have been reported in Tanzania [21], Egypt [24], Nigeria, UK, USA [25] and Portugal [13]. Dietary exposure to toxic elements in adults and young individuals has been assessed in many countries [26,27,28,29,30]. In our previous study, toxic elements in raw milk in China have been assessed [31]. However, the domestic and imported infant formulas have not been systematically investigated. Therefore, consider the scarcity, the aim of this study was to (i) determine the toxic elements (heavy metals and arsenic) contamination in infant formulas (stages 1 through 4) in Beijing, (ii) check whether these samples meet legal requirements, (iii) compare toxic elements in domestic and imported samples, (iv) evaluate the exposure to toxic elements from infant formulas and to assess the potential health risks to infants in China.

## 2. Materials and Methods

### 2.1. Sampling

A total of 93 cow milk-based infant formulas from Beijing were collected in 2019–2020 (Table 1). Among those formulas, 27 were domestic and 66 were imported. For domestic brands, Feihe, Junlebao and Yili were selected. For imported brands, Illuma, Arla, Abbott, Nestle, HiPP, Biostime, Nutricia, Friesland, Meadjhnson, Wyeth, Anmum, Karihome and a2 were selected. These samples cover large swathes of the infant formula market in Beijing. Furthermore, there were stage 1 (28), stage 2 (27), stage 3 (24) and stage 4 (14) infant formulas. Samples were sampled in supermarkets. The samples were maintained at −20 °C prior to analysis.

### 2.2. Sample Analysis

To 0.5 g infant formula sample in a polyfluoroalkoxy digestion vessel, we added 1 mL deionized water (Milli-Q, Millipore, Bedford, MA, USA), 5 mL of HNO_3_ (65%, Suprapur, Merck, Darmstadt, Germany) and 2 mL of H_2_O_2_ (30%, Suprapur, Merck, Darmstadt, Germany). Following an overnight pre-digestion at room temperature, the mixture was digested in a microwave-assisted reaction system (CEM MARs 6, Charlotte, NC, USA) according to the program shown in Table 2. Once cooled to room temperature, the digest was diluted with deionized water to 25 mL and analyzed by inductively coupled plasma mass spectrometry (ICP-MS; Agilent 7700 Series ICP-MS, Agilent Technologies, Santa Clara, CA, USA) after filtration through a 0.22-μm membrane.

We developed standard five-point calibrations for each of the toxic elements. The correlation coefficients were >0.999 and the limits of detection for Pb, As, Cr and Cd in infant formula was 0.2, 0.5, 2.0 and 0.1 μg/kg, respectively. To assess the accuracy of the method, milk powder certificate reference material (CRM, Code: GBW 10117, National Institute of Metrology, Beijing, China) were analyzed. The recovery of the elements from milk powder CRM is shown in Table 3. The recovery of these four toxic elements was 92.3%–104.3%.

### 2.3. Risk Assessment

The risk of toxic elements for infants (0.5 to 5 y of age) was assessed by calculating the estimated daily intake (EDI), target hazard quotient (THQ) and hazard index (HI). All experimental procedures for this study were approved by the ethics committee of Chinese Academy of Agricultural Sciences.

#### 2.3.1. Exposure Assessment

The exposure of infants to toxic elements from infant formula consumption was assessed using the average content of toxic elements in the test and the recommended average consumption of infant formula in China [4] according to Equation (1) [27].We compared the EDI obtained in this study with the reference dose (RfD). RfD is useful as a reference point from which to gauge the potential effects of the chemical at other doses. Usually, doses less than the RfD are not likely to be associated with adverse health risks and are therefore less likely to be of regulatory concern [29].

EDI = C × DI/BW(1)

where C is the toxic elements content in infant formula (μg/kg), DI is the daily infant formula intake (kg) and BW is body weight (kg).

#### 2.3.2. Target Hazard Quotient (THQ)

The potential chronic risk from toxic elements was expressed as THQ. THQ value was used to assess non-carcinogenic risk. THQ values < 1 indicate that consumers are unlikely to experience any adverse health effects. If the THQ value ≥ 1, there is a potential health risk. It was calculated using Equation (2)

THQ = EDI/RfD(2)

where RfD is the oral reference dose (mg/kg/d), based on 3 × 10^−4^, 4 × 10^−3^, 3 × 10^−3^ and 1× 10^−3^ for As, Pb, Cr and Cd, respectively [30,32,33,34,35].

#### 2.3.3. Health Risks of Multiple Toxic Elements

In this study, total THQ was also estimated because people usually suffered combined effects expose to several pollutants [32]. The total potential chronic risk from multiple toxic elements was expressed as a hazard index (HI), which was calculated using Equation (3). HI < 1 indicated no risk for human health [14,36].

HI = ∑THQ(3)

### 2.4. Statistical Analysis

Data analysis was performed using SPSS (IBM, Endicott, NY, USA) version 20. Data were expressed as mean ± standard error (SE). Differences in heavy metal content among samples were analyzed with an independent *t*-test. *p* < 0.05 was considered statistically significant.

## 3. Results and Discussion

### 3.1. Concentrations of Toxic Elements in Infant Formula

A total of 93 infant formula samples were sampled in China and comprehensively measured 4 toxic elements. Samples, which belonged to nine countries, were for different infant growth stages. The content of toxic elements in infant formulas were 27.38 μg/kg Cr (2.51–83.80 μg/kg), 3.32 μg/kg As (0.89–7.87 μg/kg), 0.98 μg/kg Cd (0.13–3.58 μg/kg) and 2.03 μg/kg Pb (0.36–5.57 μg/kg; Table 4 and Table 5).

The contents of Pb in this study were below the MLs established by China (150 μg/kg) [16], European Union (50 μg/kg) [18] and CAC (10 μg/kg) [17]. Furthermore, the Cd content was below the ML set by the European Union (10 μg/kg in infant formula) [18]. These results reveal the safety of infant formulas in China as a result of safety measures introduced by the Chinese government [37]. The average price was higher for imported infant formulas than for domestic infant formulas (74.56 vs. 52.50 yuan in Beijing market for 400 g infant formula) [5]. Not all families may be able to afford the high price of imported infant formulas, especially migrant workers with low income. Domestic infant formula would be an optimal choice.

The contents of Cr, As, Pb and Cd were 28.77 ± 14.69, 3.48 ± 1.41, 2.13 ± 0.96 and 0.77 ± 0.75 μg/kg, respectively, in the domestic samples and 26.71 ± 16.43, 3.24 ± 1.57, 1.99 ± 1.33 and 1.08 ± 0.77 μg/kg, respectively, in the imported samples. Even though there were no significant differences in the contents of toxic elements between the domestic and imported infant formula sample (*p* > 0.05), Cd contents were slightly lower in domestic samples.

The content of toxic elements in infant formulas reported in other studies are summarized in Table 6. The Cr content in our study was in accordance with the Cr levels reported in Tanzania (<7–53 μg/kg) [21], Saudi Arabia (37 ± 55 μg/kg) [38], Poland (<100 μg/kg) [39] but higher than the levels reported in Nigeria, UK and USA [25] and lower than in Egypt [24]. As for Pb, the contents were within the range (0.14–1,850 μg/kg) reported by other investigators [3,21,24,25,35,37,38]. The Cd contents were similar to those reported in Tanzania (<1–7 μg/kg) [21], Saudi Arabia (7 ± 5 μg/kg) [38], Poland (<10 μg/kg) [39] and Canada (0.03–1.26 μg/kg) [40].

### 3.2. Risk Assessment of Toxic Elements Infant Formula

Due to some key physiological differences between infants and adults, infants are far more vulnerable to the environmental contaminants, thus to the increased doses of exposure. In infants, some protection mechanisms such as blood-brain barrier, plasma protein binding capacity, enzymatic elimination mechanisms in the liver and kidneys and immune system are underdeveloped [41,42,43,44]. Infants are particularly sensitive to ingested contaminants due to larger specific surface area and efficient gastrointestinal absorption [21]. Infant formulas represent the main or only source of nutrients for infants when breastmilk is absent or insufficient. Therefore, the toxic elements in infant formulas, which may constitute a risk factor for the babies, is highly significant. It is essential to ensure their quality and safety.

Toxic elements is absorbed more in young people. It accumulates in soft tissues and over time in bones, for its long half-lives in blood and bone. The Panel on Contaminants in the Food Chain (CONTAM Panel) of European Food Safety Authority (EFSA) and the Joint Food and Agriculture Organization/World Health Organization Expert Committee on Food Additives (JECFA) identified developmental neurotoxicity in young children and cardiovascular effects and nephrotoxicity in adults as critical effects for risk assessment [15]. In 1987, the Provisional Tolerable Weekly Intake (PTWI) of Pb was established at 25 μg/kg bw. However, based on a dose-response analysis, the JECFA withdrew it based on a dose-response analysis [45]. In 2010, the JECFA established a provisional tolerable monthly intake (PTMI) of 25 μg/kg bw for Cd, equal to 5.8 μg/kg bw per week [46]. Inorganic arsenic (iAs) is more toxic than organic arsenic compounds [47]. Similarly, hexavalent chromium (Cr IV) is more toxic than trivalent chromium (Cr III). Therefore, we assumed that As and Cr in milk were iAs and Cr (IV). In 2011, the PTWI (2.1 μg/kg bw per day) for iAs was withdrawn by Joint FAO/WHO Expert Committee on Food Additives (JECFA) [48]. The International Programme on Chemical Safety (ICPS) established the tolerable daily intake (TDI) for Cr (VI) (0.9 μg/kg bw per day) for oral exposure [49]. Considering the absence of PTWI value for Pb and As, we used the human health risk assessment model to characterize the potential risk of toxic elements via consumption of infant formulas from Beijing. The model was suggested to calculate health risk requirements (EDI and THQ) [50,51].

The exposure to the four toxic elements from infant formula was assessed based on the mean concentration. We estimated EDI and THQ for infants between the ages of 0.5 and 5 y, taking into account infant formula intake and average body weight (Table 7). We assumed that milk was exclusively provided by infant formula. The exposure of toxic elements for boys and girls was 0.008–0.028 and 0.008–0.031 μg/kg BW/day As, 0.0061–0.0152 and 0.0062–0.0165 μg/kg BW/day Pb, 0.073–0.256 and 0.074–0.278 μg/kg BW/day Cr and 0.0025–0.0085 and 0.0025–0.0090 μg/kg BW/day Cd, respectively. The EDIs of As, Pb, Cr and Cd were lower than RfD references of 0.3, 4.0, 3.0, 1.0 and 0.5 μg/kg BW/day [30,32,33,34,35]. The exposure of As, Pb, Cr and Cd were lower than the corresponding RfD values.

The THQ values for boys and girls were 0.024–0.085 and 0.025–0.093 for As, 0.0015–0.0043 and 0.0015–0.0046 for Pb, 0.024–0.085 and 0.0025–0.0093 for Cr, 0.0025–0.0083 and 0.0025–0.0090 for Cd, respectively. The THQ reported by other studies are shown in Table 8. The exposure level of Pb was comparable to that reported in Italy [26] and lower than that reported in Nigeria, Turkey, Ethiopia and Iran [15,27,28,29]. The exposure of Cr was slightly lower than that reported in Italy [26], Turkey [28] and Ethiopia [29]. As for Cd, the exposure in this study was low compared to previous studies. The THQ of As, Cr, Cd and Pb was lower in this study than in Mexico.

**Figure 1:**
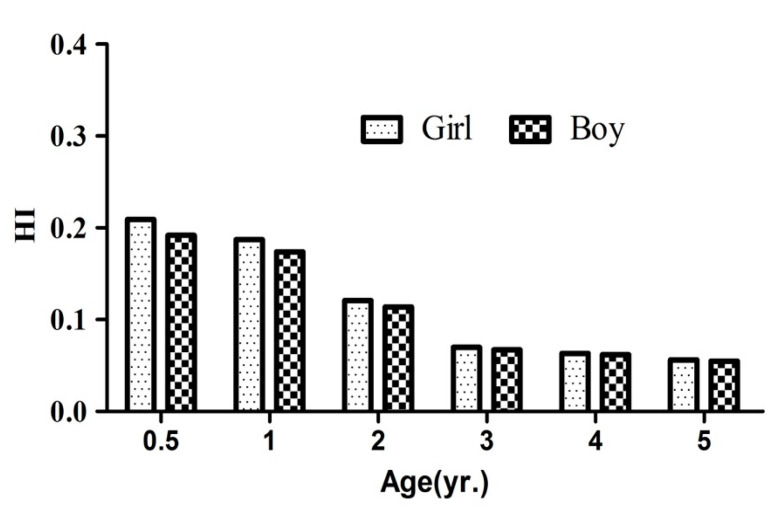
Hazard index (HI) of boys and girls of different age.

The HI values were calculated from total THQ corresponding to each body weight at different age. The HI values are shown in Figure 1. The HI values were 0.055–0.192 for boys and 0.056–0.209 for girls. The HI value was higher for girls than boys, due to their lower body weight. The HI values were inversely associated with age and were lower than the established criteria 1. The non-carcinogenic risk value were in the safe range, indicating that exposure of As, Pb, Cr and Cd from infant formulas do not represent a health risk in China. However, food producers should try their best to reduce the levels of toxic elements in infant formula [16] considering the immunodeficiency and intestinal hypoplasia for young people.

## 4. Conclusions

A total of 93 infant formula samples of domestic and imported brands obtained from the Chinese market were analyzed for As, Cr, Pb and Cd by ICP-MS. The content of Pb in the samples was below the ML set by China, CAC and the European Union and the content of Cd was below the ML set by the European Union. Imported brands, which were more costly, did not have significantly lower heavy metal contents compared to domestic brands. Dietary exposure of As, Cr and Cd in infants (0.5–5 y) was below the corresponding RfD values. The THQ and HI values of these toxic elements were <1. This study suggested that the presence of toxic elements in infant formulas in Beijing, China should not pose a non-carcinogenic risk for infant health. Domestic infant formulas are preferred for infant when breastmilk is absent or insufficient. Further study is necessary to estimate macro elements, trace elements and other toxic elements in infant formulas in Beijing, to compare nutritional values not only the potential risk.

## Figures and Tables

**Table 1:** Sample characteristics.

Country	Stage			
	1	2	3	4
China	8	8	8	3
Ireland	2	2	2	2
Denmark	2	2	1	0
Germany	2	2	1	0
France	1	1	1	0
Netherlands	9	9	8	9
Switzerland	1	1	1	0
New Zealand	3	3	2	0
Total	28	27	24	14

**Table 2:** Microwave-assisted digestion procedure.

Temperature (°C)	Gradient Temperature Time (min)	Holding Time (min)
90	10	5
140	10	10
190	10	20

**Table 3:** Quality control of certified reference material, milk powder.

Toxic Elements	Certified Reference Material		
	Certified Values (μg/kg)	Observed Values (μg/kg)	Recovery (%)
Cr	1900	1753.70	92.3
As	200	192.40	96.2
Cd	111	115.77	104.3
Pb	416	397.28	95.5

**Table 4:** Heavy toxic elements (μg/kg) in infant formulas of different stages.

Stage	N	Cr		As		Cd		Pb	
		Mean	Range	Mean	Range	Mean	Range	Mean	Range
1	28	27.09	2.51–67.70	3.00	0.89–7.87	0.88	0.16–3.53	1.61	0.42–4.82
2	27	26.71	8.12–58.33	3.57	1.09–6.34	1.01	0.18–2.62	2.19	0.81–5.45
3	24	27.39	4.81–83.80	3.39	1.29–5.53	1.03	0.23–3.58	2.09	0.36–3.60
4	14	30.62	5.68–56.15	3.37	1.35–5.54	1.04	0.13–3.17	2.55	0.62–5.75
Total	93	27.38	2.51–83.80	3.32	0.89–7.87	0.98	0.13–3.58	2.03	0.36–5.75

**Table 5:** Toxic elements content (μg/kg) in domestic and imported infant formulas.

Toxic Elements	Domestic (*n* = 27)		Imported (*n* = 66)		Maximum Level [16,17,18]		
	Mean ± SD	Range	Mean ± SD	Range	China	EU	CAC
Cr	28.77 ± 14.69	4.48–58.33	26.71 ± 16.43	2.51–83.80	— ^a^	— ^a^	— ^a^
As	3.48 ± 1.41	0.89–5.19	3.24 ± 1.57	0.98–7.87	— ^a^	— ^a^	— ^a^
Cd	0.77 ± 0.75	0.13–3.17	1.08 ± 0.77	0.16–3.58	— ^a^	10	— ^a^
Pb	2.13 ± 0.96	0.63–4.08	1.99 ± 1.33	0.36–5.75	150	50	10

^a^ Not mentioned in reference.

**Table 6:** Toxic elements content in infant formulas from different countries.

Year	Country	N	Toxic Elements	Mean (μg/kg)	Range (μg/kg)	Reference
— ^a^	Tanzania	8	Cr	— ^a^	<7–53	[21]
			Cd	— ^a^	<1–7	
			Pb	— ^a^	<10–50	
— ^a^	Egypt	50	Pb	791 ± 57	450–1850	[24]
			Cr	322 ± 39	100–1450	
2009	Saudi Arabia	19	Cd	7 ± 5	— ^a^	[38]
			Cr	37 ± 55	— ^a^	
			Pb	18 ± 2	— ^a^	
			Cd	<10	— ^a^	
			Cr	<100	— ^a^	
			Pb	<5	— ^a^	
1999	Canada	57	Pb	0.9	0.14–2.46	[40]
			Cd	0.23	0.03–1.26	
2017	Ethiopia	— ^a^	Cd	ND ^b^	— ^a^	[29]
			Pb	46	— ^a^	
2000	Nigeria	6	Cd	ND ^b^	— ^a^	[25]
			Cr	6 ± 3	— ^a^	
			Pb	0.4 ± 1.0	— ^a^	
	UK	21	Cd	ND ^b^	— ^a^	
			Cr	5 ± 5	— ^a^	
			Pb	0.8 ± 1.7	— ^a^	
	USA	15	Cd	ND ^b^	— ^a^	
			Cr	7 ± 9	— ^a^	
			Pb	ND ^b^	— ^a^	
2007–2008	Portugal	19	Hg	0.64	0.15–0.85	[13]
— ^a^	Spain	7	Pb	2570 ± 6210	<LOD ^c^–17240	[3]
2019	China	93	Cr	27.38	2.51–83.80	This study
			As	3.32	0.89–7.87	
			Cd	0.98	0.13–3.58	
			Pb	2.03	0.36–5.75	

^a^ Not mentioned in reference; ^b^ Not detected; ^c^ Limit of detection.

**Table 7:** Estimated daily intake (EDI) and target hazard quotient (THQ) of Cr, As, Cd, Pb in infants and children due to consumption of infant formula.

Age	Gender	Body Weight (kg) [52]	EDI				THQ			
			Cr	As	Cd	Pb	Cr	As	Cd	Pb
0.50	Girl	7.30	0.278	0.031	0.0090	0.0165	0.093	0.103	0.0090	0.0041
	Boy	7.93	0.256	0.028	0.0083	0.0152	0.085	0.095	0.0083	0.0038
1	Girl	8.95	0.224	0.030	0.0085	0.0184	0.075	0.100	0.0085	0.0046
	Boy	9.65	0.208	0.028	0.0078	0.0170	0.069	0.092	0.0078	0.0043
2	Girl	11.48	0.150	0.019	0.0057	0.0115	0.050	0.062	0.0057	0.0029
	Boy	12.15	0.142	0.018	0.0053	0.0108	0.047	0.059	0.0053	0.0027
3	Girl	13.85	0.087	0.011	0.0033	0.0066	0.029	0.036	0.0033	0.0017
	Boy	14.34	0.084	0.010	0.0032	0.0064	0.028	0.035	0.0032	0.0016
4	Girl	16.07	0.084	0.009	0.0028	0.0070	0.028	0.031	0.0028	0.0017
	Boy	16.34	0.082	0.009	0.0028	0.0069	0.027	0.030	0.0028	0.0017
5	Girl	18.22	0.074	0.008	0.0025	0.0062	0.025	0.027	0.0025	0.0015
	Boy	18.34	0.073	0.008	0.0025	0.0061	0.024	0.027	0.0028	0.0015

**Table 8:** EDI and THQ from other countries.

Country	Milk Style	Metal	Stage	EDI (μg/kg bodyweight/day)	THQ	Reference
Italy	Infant formula	Cr	Infant	0.51	— ^a^	[26]
		Cd		0.02	— ^a^	
		Pb		0.01	— ^a^	
Nigerian	Infant formula	Cd	Infant	0.2	— ^a^	[27]
		Pb		1.3	— ^a^	
		Cr		20.6	— ^a^	
Turkey	Infant formula	Pb	Infant	0.10–0.15	— ^a^	[28]
		Cd	Infant	0.06–0.10	— ^a^	
		Cr	Infant	0.56–0.98	— ^a^	
UK	Infant formula	Cr	Infant	5.7 ^b^	— ^a^	[25]
USA		Cr	Infant	7.7 ^b^	— ^a^	
Nigeria		Cr	Infant	7.35 ^b^	— ^a^	
Ethiopia	Infant formula	Pb	Infant	0.614–1.064	— ^a^	[29]
Egypt	Powder	Pb	Adult	2.26	— ^a^	[24]
		Cd		0.91	— ^a^	
Mexico	Raw milk	Pb	Children	— ^a^	0.024–0.034	[27]
		Cd		— ^a^	0.041–0.046	
		Cr		— ^a^	0.024–0.025	
		As		— ^a^	2.93–3.05	
Iran	Raw milk	Pb	Adult	0.11	— ^a^	[15]
		Cd		0.01	— ^a^	
China	Infant formula	As	Infant	0.008–0.031	0.027–0.103	This study
		Pb		0.0061–0.0170	0.0015–0.0046	
		Cr		0.073–0.256	0.024–0.093	
		Cd		0.0025–0.0090	0.0025–0.0090	

^a^ Not mentioned in reference; ^b^ μg/day.

## References

[1] WHO Breastfeeding. https://www.who.int/health-topics/breastfeeding#tab=tab_1.

[2] Yang Z., Lai J., Yu D., Duan Y., Pang X., Jiang S., Bi Y., Wang J., Zhao L., Yin S. (2016). Breastfeeding rates in China: A cross-sectional survey and estimate of benefits of improvement. Lancet.

[3] Navarro-Blasco I., Alvarez-Galindo J.I. (2005). Lead levels in retall samples infant formulae and their contribution to dietary intake of infants. Food Addit. Contam..

[4] Chinese Nutrition Society (2018). Dietary Guidelines for Chinese Women and Children (2016).

[5] Editorial Board Member of China Dairy Yearbook (2019). China Dairy Industry Yearbook.

[6] World Health Organization(WHO) (2020). IARC Monographs on the Identification of Carcinnogenic Hazards to Humans.

[7] Pandya C.D., Pillai P.P., Gupta S.S. (2010). Lead and cadmium co-exposure mediated toxic insults on hepatic steroid metabolism and antioxidant system of adult male rats. Biol. Trace Elem. Res..

[8] Norwood W.P., Borgmann U., Dixon D.G., Wallace A. (2003). Effects of metal mixtures on aquatic biota: A review of observations and methods. Hum. Ecol. Risk Assess..

[9] Kortenkamp A. (2007). Ten years of mixing cocktails: A review of combination effects of endocrine-disrupting chemicals. Environ. Health Perspect..

[10] Su H., Li Z., Kenston S.S.F., Shi H., Wang Y., Song X., Gu Y., Barber T., Aldinger J., Zou B. (2017). Joint toxicity of different heavy metal mixtures after a short-term oral repeated-administration in rats. Int. J. Environ. Res. Public Health.

[11] Daneman R., Rescigno M. (2009). The Gut Immune Barrier and the Blood-Brain Barrier: Are They So Different?. Immunity.

[12] Weström B., Arévalo Sureda E., Pierzynowska K., Pierzynowski S.G., Pérez-Cano F.J. (2020). The Immature Gut Barrier and Its Importance in Establishing Immunity in Newborn Mammals. Front. Immunol..

[13] Martins C., Vasco E., Paixão E., Alvito P. (2013). Total mercury in infant food, occurrence and exposure assessment in Portugal. Food Addit. Contam. Part B Surveill..

[14] Castro-González N.P., Calderón-Sánchez F., Pérez-Sato M., Soní-Guillermo E., Reyes-Cervantes E. (2019). Health risk due to chronic heavy metal consumption via cow’s milk produced in Puebla, Mexico, in irrigated wastewater areas. Food Addit. Contam. Part B Surveill..

[15] Norouzirad R., González-Montaña J.R., Martínez-Pastor F., Hosseini H., Shahrouzian A., Khabazkhoob M., Ali Malayeri F., Moallem Bandani H., Paknejad M., Foroughi-nia B. (2018). Lead and cadmium levels in raw bovine milk and dietary risk assessment in areas near petroleum extraction industries. Sci. Total Environ..

[16] National Health and Family Planning Commission (2017). National Medical Products Administration of China National Food Safety Standard—Limits of Contaminants in Food. GB 2762-2017.

[17] Codex Alimentarius Commission (2019). General Standard for Contaminants and Toxins in Food and Feed.

[18] EC (2006). Regulation (EC) No 1881/2006 of 19 December 2006 setting maximum levels for certain contaminants in foodstuffs. Off. J. Eur. Union.

[19] ATSDR The Agency for Toxic Substances and Disease Registry’s 2019 Substance Priority List.

[20] State Administration for Markt Regulation (2019). Food Safety Supervision and Sampling Plan in 2019 in China. http://www.samr.gov.cn/spcjs/cjjc/qtwj/201902/t20190226_291363.html.

[21] Sager M., McCulloch C.R., Schoder D. (2018). Heavy metal content and element analysis of infant formula and milk powder samples purchased on the Tanzanian market: International branded versus black market products. Food Chem..

[22] Ljung K., Palm B., Grandér M., Vahter M. (2011). High concentrations of essential and toxic elements in infant formula and infant foods—A matter of concern. Food Chem..

[23] Schilmann K. (1990). The toxicological extimation of the heavy metal content (Cd, Hg, Pb) in food for infants and small children. Z. Ernahr..

[24] Salah F.A.A.E., Esmat I.A., Bayoumi M.A. (2013). Heavy metals residues and trace elements in milk powder marketed in Dakahlia Governorate. Int. Food Res. J..

[25] Ikem A., Nwankwoala A., Odueyungbo S., Nyavor K., Egiebor N. (2002). Levels of 26 elements in infant formula from USA, UK, and Nigeria by microwave digestion and ICP-OES. Food Chem..

[26] Bargellini A., Venturelli F., Casali E., Ferrari A., Marchesi I., Borella P. (2018). Trace elements in starter infant formula: Dietary intake and safety assessment. Environ. Sci. Pollut. Res..

[27] Iwegbue C.M.A., Nwozo S.O., Overah L.C., Nwajei G.E. (2010). Survey of trace element composition of commercial infant formulas in the nigerian market. Food Addit. Contam. Part. B Surveill..

[28] Sipahi H., Eken A., Aydın A., Şahin G., Baydar T. (2015). Safety assessment of essential and toxic metals in infant formulas. Turk. J. Pediatr..

[29] Eticha T., Afrasa M., Kahsay G., Gebretsadik H. (2018). Infant Exposure to Metals through Consumption of Formula Feeding in Mekelle, Ethiopia. Int. J. Anal. Chem..

[30] Castro Gonzalez N.P., Moreno-Rojas R., Calderón Sánchez F., Moreno Ortega A., Juarez Meneses M. (2017). Assessment risk to children’s health due to consumption of cow’s milk in polluted areas in Puebla and Tlaxcala, Mexico. Food Addit. Contam. Part. B Surveill..

[31] Qu X.Y., Zheng N., Zhou X.W., Li S.L., Wang J.Q., Zhang W.J. (2018). Analysis and Risk Assessment of Seven Toxic Element Residues in Raw Bovine Milk in China. Biol. Trace Elem. Res..

[32] US EPA (1993). Reference Dose (RfD): Description and Use in Health Risk Assessments. https://www.epa.gov/iris/reference-dose-rfd-description-and-use-health-risk-assessments.

[33] US EPA (1998). Chromium (VI).

[34] US EPA (1991). Arsenic (Inorganic).

[35] (1989). Human Health Evaluation Manual (Part A). EPA Risk Assessment Guidance for Superfund.

[36] Khan K., Khan H., Lu Y., Ihsanullah I., Nawab J., Khan S., Shah N.S., Shamshad I., Maryam A. (2014). Evaluation of toxicological risk of foodstuffs contaminated with heavy metals in Swat, Pakistan. Ecotoxicol. Environ. Saf..

[37] Li S., Min L., Wang P., Zhang Y., Zheng N., Wang J. (2017). Occurrence of aflatoxin M1 in pasteurized and UHT milks in China in 2014–2015. Food Control.

[38] Al Khalifa A.S., Ahmad D. (2010). Determination of key elements by ICP-OES in commercially available infant formulae and baby foods in Saudi Arabia. Afr. J. Food Sci..

[39] Chajduk E., Pyszynska M., Polkowska-Motrenko H. (2018). Determination of trace elements in infant formulas available on polish market. Biol. Trace Elem. Res..

[40] Dabeka R., Fouquet A., Belisle S., Turcotte S. (2011). Lead, cadmium and aluminum in Canadian infant formulae, oral electrolytes and glucose solutions. Food Addit. Contam. Part A.

[41] Kunter İ., Hürer N., Gülcan H.O., Öztürk B., Doğan İ., Şahin G. (2017). Assessment of Aflatoxin M1 and Heavy Metal Levels in Mothers Breast Milk in Famagusta, Cyprus. Biol. Trace Elem. Res..

[42] Koller K., Brown T., Spurgeon A., Levy L. (2004). Recent developments in low-level lead exposure and intellectual impairment in children. Environ. Health Perspect..

[43] Chance G.W. (2001). Environmental contaminants and children’s health: Cause for concern, time for action. Paediatr. Child Health.

[44] Pohl H.R., Hibbs B.F. (1996). Breast-feeding exposure of infants to environmental contaminants—A public health risk assessment viewpoint: Chlorinated dibenzodioxins and chlorinated dibenzofurans. Toxicol. Ind. Health.

[45] JECFA (2011). Lead. Safety Evaluation of Certain Food Additives and Contaminants. Seventy-Third Meeting of the Joint FAO/WHO Expert Committee on Food Additives.

[46] JECFA (2011). Cadmium. Safety Evaluation of Certain food Additives and Contaminants. Seventy-Third Meeting of the Joint FAO/WHO Expert Committee on Food Additives.

[47] EFSA (2014). Dietary exposure to inorganic arsenic in the European population. EFSA J..

[48] World Health Organization (2011). Evaluation of Certain Contaminants in Food.

[49] IPCS (2009). Principles and methods for the risk assessment of chemicals in food. International Programme on Chemical Safety. Environ. Health Criteria.

[50] Hashemi M., Sadeghi A., Saghi M., Aminzare M., Raeisi M., Rezayi M., Sany S.B.T. (2019). Health Risk Assessment for Human Exposure to Trace Metals and Arsenic via Consumption of Hen Egg Collected from Largest Poultry Industry in Iran. Biol. Trace Elem. Res..

[51] Bortey-Sam N., Nakayama S.M.M., Ikenaka Y., Akoto O., Baidoo E., Yohannes Y.B., Mizukawa H., Ishizuka M. (2015). Human health risks from metals and metalloid via consumption of food animals near gold mines in Tarkwa, Ghana: Estimation of the daily intakes and target hazard quotients (THQs). Ecotoxicol. Environ. Saf..

[52] WHO (2006). Lengthheight-for-age, weight-for-age, weight-for-length, weight-for-height and body mass index-for-age Methods and development. WHO Child Growth Standards WHO.

